# Utility of SOX2 and Livin Co-Expression in the Prognosis of Bladder Cancer With Bilharzial and Non-Bilharzial Bladder Status

**DOI:** 10.14740/wjon942w

**Published:** 2015-10-26

**Authors:** Mariana Fathy Gayyed, Ehab Rifat Tawfiek

**Affiliations:** a Department of Pathology, Faculty of Medicine, Minia University, Minia, Egypt; b Department of Urology, Faculty of Medicine, Minia University, Minia, Egypt

**Keywords:** SOX2, Livin, TCC, SCC, Immunohistochemistry

## Abstract

**Background:**

The aim of this study was to investigate the expression of SOX2, a key transcription factor and livin, an apoptotic inhibitor in bladder transitional cell carcinoma (TCC) and squamous cell carcinoma (SCC). Moreover, their prognostic significance was assessed.

**Methods:**

The expressions of SOX2 and livin in 82 TCC and 35 SCC cases were detected by immunohistochemistry.

**Results:**

SOX2 and livin were over-expressed in tumor tissues as compared to the corresponding adjacent non-neoplastic tissues. SOX2 and livin expressions were significantly associated with high tumor grade (P = 0.002 and P = 0.007, respectively) and high tumor stage (P = 0.027 and P = 0.033, respectively). No significant correlation was found between tumor and other clinicopathological factors such as age, gender and schistosomal status. Univariate analysis revealed that TCC and SCC patients with high SOX2 or livin expressions were significantly related to overall survival (P < 0.001, P = 0.025 for TCC patients and P = 0.041, P = 0.021 for SCC patients, respectively). Multivariate survival analysis further demonstrated that SOX2 expression was an independent prognostic factor for TCC patients (P = 0.015).

**Conclusions:**

SOX2 and livin may contribute to the progression of bladder carcinoma. SOX2/livin pathway regulates cancer stem cell survival so it could be targeting as an effective therapeutic strategy for cancer treatment.

## Introduction

Bladder cancer, the most common urinary tract system cancer, is the sixth most prevalent cancer in men worldwide and the fifth most frequent cancer in men in the western countries [1]. In Egypt, it is the second common malignancy among Egyptian males [2].

Transitional cell carcinoma (TCC) represents about 90% of the bladder cancer cases while the other types including squamous cell carcinoma (SCC), adenocarcinoma and other rare types comprise the remaining 10% of cases [3]. Previous research has reported a significant decrease in SCC in Egypt, although the overall bladder cancer incidence in Egypt has remained steady due to an increase in TCC over the past 30 years [4].

TCC is further classified into papillary and invasive carcinomas [1]. Low grade papillary carcinomas have a better prognosis than high grade papillary carcinomas which have a higher tendency to become invasive. The invasive TCC includes non-muscle invasive bladder cancer (non-MIBC) (Ta-T1) and MIBC (T2-T3). About 20-40% of non-MIBC has been progressed into MIBC within 5 years depending on some risk factors such as high grade, multiplicity, large tumor size (> 3 cm), and concomitant carcinoma *in situ* (CIS) stage. Till now, radical cystectomy is the treatment of choice in case of MIBC [5].

In Egypt, the major risk factors for TCC are considered occupational exposure and alterations in tumor suppressor genes [6, 7]. Unlike TCC, the main risk factors for SCC are exposure to infectious agent namely Schistosoma hematobium, a trematode that induces its effect via deposition of the bilharzial ova mainly in the submucosa of the urinary bladder and lower ureters, and to a lesser extent in the upper ureters and kidneys. Bilharzial ova deposited in the submucosa result in different urothelial changes with some pre-cancerous lesions, e.g. urothelial hyperplasia, cystitis cystica, cystitis glandularis, squamous metaplasia and CIS. Schistosoma hematobium infection predisposes also to increased bacterial infections that eventually lead to SCC [8]. One of the Egyptian studies reported that, in 1980, 22% of Egyptian bladder cancer cases were diagnosed as TCC and 78% were diagnosed as SCC. In 2005, that ratio was nearly the opposite with 73% of bladder cancers diagnosed as TCC and 28% diagnosed as SCC. Such decline in SCC cases has been attributed to decrease in the Schistosoma hematobium infection due to public health interventions and changes in the Nile river system [4].

Bladder carcinogenesis is a multistep process involving the cancer stem cells (CSCs) hypothesis which stated that tumor might be initiated and maintained by a population of cells with stem cell-like characters [9]. CSCs have been identified in many tumors of different tissues [10-12]. This cellular population exhibits gene expression signatures closely related to embryonic stem cells [9].

SOX2, a member of the SOX family (SRY-related high mobility group box), is a key transcription factor involved in maintaining the pluripotency, self-renewal and differentiation of embryonic stem cell. SOX2 gene is located at chromosome 3q26.33 [13].

Recent studies have demonstrated that SOX2 has a potential function in tumorigenesis. SOX2 over-expression is potentially involved in prognosis as it has been highly expressed in tumors displaying lower degrees of differentiation [14]. SOX2 has been found to be deregulated in several human tumors including pancreatic, esophageal, colonic, cervical, ovarian, prostatic, breast, lung, gastric carcinomas and glioma [15-24].

Another mechanism of bladder carcinogenesis is disturbed apoptosis. Apoptosis plays a vital role in morphogenesis, cell turnover and elimination of harmful cells. An inhibition of apoptosis may present a survival advantage on malignant cells harboring genetic alterations and thus promote cancer progression [25]. The main key in apoptosis is the proteolytic activation of the caspases, a class of cysteine aspartyl-specific proteases. Initiator caspases cleave executive caspases which in turn degrade a number of intracellular protein substrates resulting in the characteristic morphological hallmarks of apoptosis. These caspase activities are inhibited by the inhibitors of apoptosis proteins (IAPs) family. Until now, eight human IAPs have been identified, including livin, survivin, c-IAP1, c-IAP2, NAIP, XIAP, ILP-2 and BRUCE [26]. The IAPs let cancer cells resistant to apoptotic stimulation. Members of IAPs have been expressed in many cancers. They are associated with poor prognosis and resistance to radiotherapy and chemotherapy [27].

Livin was recently identified to be a novel anti-apoptotic gene. Livin contains a single copy of a baculovirus IAP repeat (BIR) as well as a ring-type zinc finger domain. The livin gene is located on chromosome 20 at band q13. This gene has two transcript variants (isoform α and isoform β), which have variable antiapoptotic properties [28].

Livin is enrolled to death receptor signaling complexes, where it inhibits apoptosis mainly by interacting with caspase 3. This protein in turn activates caspases 6 and 7. The caspase 3 itself is activated by caspases 8, 9 and 10. It plays a central role in the execution phase of cell apoptosis [29]. Livin expression is over-expressed in most of human cancers such as lung and bladder cancers, hematological malignancies and melanoma [30-33].

Until now, no study has assessed the relationship between the expressions of SOX2 and livin in bladder cancer. Therefore, the aim of this study was to explore the role of SOX2 and livin in bladder TCC and SCC by immunohistochemical assay and to illustrate if there was a possible correlation between both of these markers with each other as well as with the clinicopathological factors.

## Material and Methods

### Patients and tissue specimens

This retrospective study included 117 surgically resected samples from patients suffering from primary bladder tumors. The patients referred to the Department of Pathology, Minia University Hospital and Minia Oncology Center, Minia, Egypt during the period from January 2010 to December 2014. Patients’ age ranged from 44 to 72 years. They were 101 males and 16 females. All patients had transurethral biopsies taken from the tumor for histopathological evaluation, followed by the appropriate treatment, e.g. transurethral resection (TUR), partial or radical cystectomy. The latter specimens were included to better assess the degree of invasion. Twenty samples of the non-neoplastic adjacent mucosa were included for comparison. This work was covered by the approval of the ethics committee of both Minia University and Minia Oncology Center.

### Histopathological evaluation

All the specimens were fixed in 10% formalin, embedded in paraffin wax and stained with hematoxylin and eosin (H&E). The H&E stained sections were examined to confirm the histopathological type, tumor grade and stage. The tumor grade was classified according to the WHO and tumor invasion was evaluated according to American Joint Committee on Cancer [34, 35]. Examination revealed TCC in 82 cases and SCC in 35 cases. Bilharzial status was evaluated by detection of bilharzial ova in tumor tissue or adjacent non-neoplastic tissue or from the clinical data associated with the patients’ records.

### Immunohistochemical assay

Immunostaining was done by using Novacastra peroxidase detection system. The procedure applied was as follow: all samples were sectioned at 4 µm and subsequently deparaffinized in xylene and rehydrated through descending grades of ethyl alcohol. Endogenous peroxidase activity was blocked by peroxidase block for 10 min. Antigen retrieval was carried out by microwave, 700 W in sodium citrate buffer (0.01 M, pH 6.0) for 20 min. The sections were incubated overnight at 4 °C in a humidity chamber with primary antibodies for rabbit polyclonal anti-SOX2 antibody, ready to use (catalog no. E18601; Spring Bioscience, CA, USA,) and rabbit polyclonal anti-livin antibody, at the dilution of 1:50 (catalog no. ab182771; Abcam Inc., Cambridge, UK). The tissue sections were then incubated at room temperature for 30 min with the biotinylated secondary antibody followed by streptavidin-HRP. The bound antibodies were visualized using diaminobenzidine tetrahydrochloride (DAB) chromogen and substrate buffer, counterstained by hematoxylin and the sections were then dehydrated through ascending grades of ethanol, mounted by distyrene, plasticizer and xylene (DPX) and finally cover-slipped. Between all incubations, sections were washed with phosphate-buffered saline (PBS). Negative controls were prepared by omitting the primary antibody with the use of PBS instead. Positive controls used were normal skin tissue for SOX2 and colon cancer tissue for livin.

### Immunohistochemical evaluation

All immunostained tissue sections were evaluated blindly regardless the clinicopathological data of the patients. For SOX2 immunostaining, a scoring system was adapted using both the percentage and the intensity of the positively stained tumor cells. The score representing the percentage of the positive tumor cells was assigned (0: negative; 1: < 25%; 2: 25-50%; 3: 50-75%; and 4: > 75%). Next, the intensity of staining of positive tumor cells was graded using 0 - 3 scale (0: negative; 1: weak; 2: moderate; and 3: strong). The percentage and intensity scores were added to obtain a final score, which ranged from 0 to 7. The specimens were then categorized into two groups according to the overall scores: low expression: < 4 scores and high expression: 4 - 7 scores. For livin immunostaining, the mean percentage of positive tumor cells was determined in at least five areas at 400-fold magnification. Accordingly, the patients were classified into two categories. High expression was defined as > 10% positively stained tumor cell and low expression if cytoplasmic staining of tumor cells was ≤ 10%. The normal/non-neoplastic bladder mucosa cells were scored for SOX2 and livin in the same method as the tumor cells.

### Statistical analysis

Statistical analysis was performed using the SPSS software version 17 (SPSS, Chicago, IL, USA) program package. The two-sided Chi-square (χ^2^) and Fisher’s exact tests were used to compare the association of SOX2 or livin expressions with clinicopathological data in TCC and SCC. Student’s *t*-test was used to compare means of patients’ ages as a continuous variable in TCC and SCC cases. P ≤ 0.05 was considered statistically significant. Overall survival (OS) was calculated from the date of initial diagnosis until last follow-up or death. Patients who were alive at last contact were treated as censored for OS analysis. Univariate and multivariate Cox proportional hazards regression analyses were performed to estimate the impact of expression of each marker on OS in these patients. Survival curves were constructed using Kaplan-Meier method. Log-rank test was used to compare the survival curves. The significance level used was P < 0.05.

## Results

### Clinicopathological data

A summary of the clinicopathological features for the cases included in this study was displayed in Table 1. In this series, a total of 117 patients with primary bladder cancer were included, from whom 70.1% were TCC, and 29.9% were SCC. The patients’ age in SCC cases was significantly lower than in TCC cases (P = 0.003). The mean patients’ ages in SCC and TCC were 53.7 ± 5.71 years and 59.12 ± 6.93 years, respectively. Male predominance in both SCC and TCC was noted with male/female ratios of 6.45:1 and 6:1, respectively. Bilharziasis was evidenced in 63/117 (53.8%) of patients. Bilharziasis associated TCC cases were significantly lower than Bilharziasis associated SCC cases (P = 0.029). According to the state of muscle invasion, all SCC cases and 76.8% of TCC cases showed muscle invasion (pT2-T3) at the time of diagnosis.

**Table 1:** Patients’ Clinicopathological Features

Clinicopathological features	TCC (n = 82/117) (70.1%)		SCC (n = 35/117) (29.9%)		P-value
Age					
≤ 50 years	12 (14.6)		14 (40)		0.003
> 50 years	70 (85.4)		21 (60)		
Gender					
Male	71 (86.6)		30 (85.7)		0.604
Female	11(13.4)		5 (14.3)		
Schistosomal status					
Negative	43 (52.4)		11 (31.4)		0.029
Positive	39 (47.6)		24 (68.6)		
Grade					
	Low	49 (59.8)	GI	12 (34.3)	< 0.001
	High	33 (40.2)	GII	16 (25.7)	
			GIII	7 (20)	
T stage					
Ta	11 (13.4)		0 (0)		0.001
T1	19 (23.2)		7 (20)		
T2	29 (35.4)		19 (54.7)		
T3	23 (28)		9 (25.7)		

P-value < 0.05 is considered significant. TCC: transitional cell carcinoma; SCC: squamous cell carcinoma.

### Expression of SOX2 and livin in non-neoplastic and neoplastic bladder tissues

**Figure 1:**
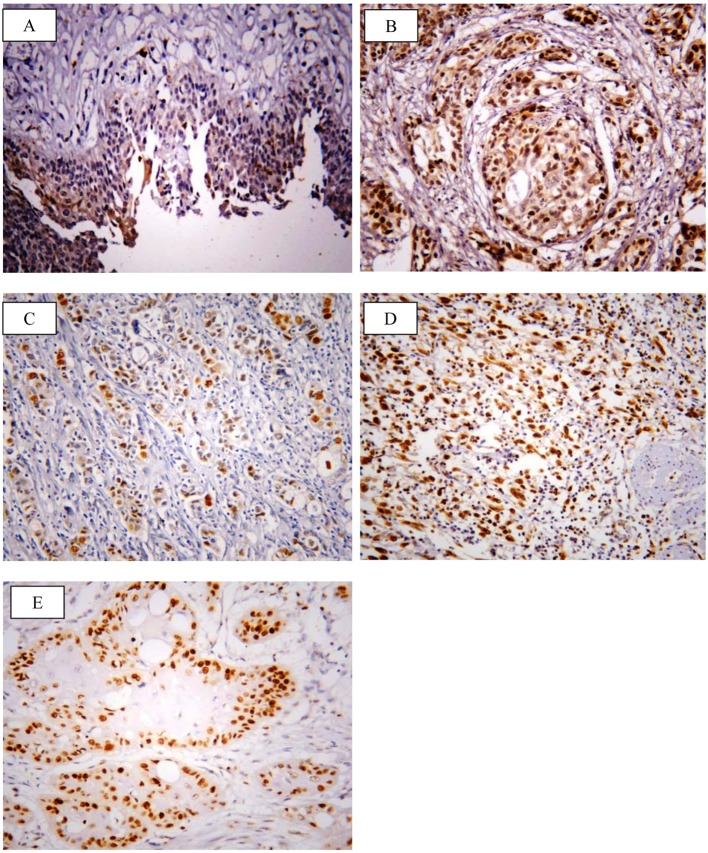
Representative immunohistochemical staining of SOX2 in human bladder cancer. (A) SOX2 showed negative/low expression in non-neoplastic bladder tissue. (B) High expression in high grade TCC. (C) Low expression in non-muscle invasive TCC. (D) High expression in muscle invasive TCC. (E) High expression in SCC (× 400; counterstained with hematoxylin).

**Figure 2:**
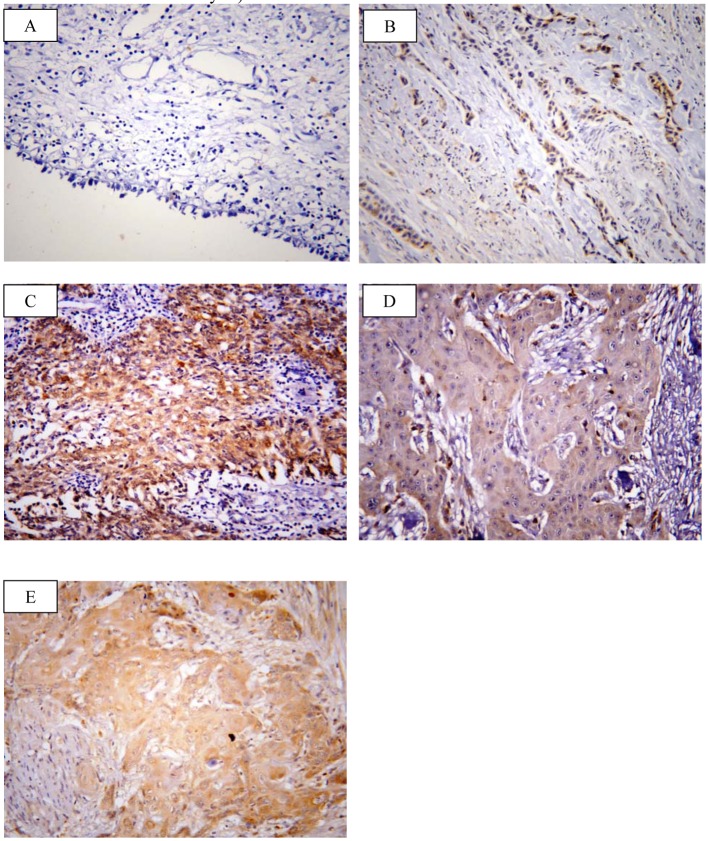
Representative immunohistochemical staining of livin in human bladder cancer. (A) Livin showed negative expression in non neoplastic bladder tissue. (B) Low expression in low grade TCC. (C) High expression in high grade TCC. (D) Low expression in SCC grade I. (E) High expression in SCC grade II (× 400; counterstained with hematoxylin).

SOX2 brown staining was localized mainly in the nucleus and to lesser extent in the cytoplasm of cells (Fig. 1A-D); however, livin immunostaining was detected in the cytoplasm of the examined cells (Fig. 2A-C). SOX2 immunostaining was negative/low expression in non-neoplastic bladder tissues. However, SOX2 high expression was found in 68/117 (58.1%) of tumor tissues. Livin immunoreactivity was undetectable in non-neoplastic bladder tissues. However, livin cytoplasmic expression was positive in 75/117 (64.1%) of cases.

TCC cases showed lower SOX2 expression (45/82, 54.9%) than SCC cases (23/35, 65.7%) (P = 0.189). Similarly, TCC cases showed positivity for livin in 51/82 (62.2%) cases which was lower than that detected in SCC cases, 24/35 (68.6%) of cases (P = 0.330).

For all examined cases (117 cases), the expressions of SOX2 and livin were significantly associated with high tumor grade (P = 0.002 and P = 0.007, respectively) and high tumor stage (P = 0.027 and P = 0.033, respectively). No significant association was found between SOX2 or livin immunoreactivity and other clinicopathological factors such as age, gender and bilharzial status.

### Expression of SOX2 and livin in TCC

Concerning TCC, the correlation between SOX2 and livin expressions and the clinicopathological data were summarized in Table 2.

**Table 2:** Relation of SOX2 and Livin Expressions With Clinicopathological Features of Bladder TCC (82 Cases)

Clinicopathological features	SOX2			Livin		
	Low (%)	High (%)	P-value	Low (%)	High (%)	P-value
Age						
≤ 50 years	6 (50)	6 (50)	0.476	7 (58.3)	5 (41.7)	0.104
> 50 years	31 (44.3)	39 (55.7)		14 (34.3)	46 (65.7)	
Gender						
Male	34 (47.9)	37 (52.1)	0.171	26 (37.1)	44 (62.9)	0.502
Female	3 (27.3)	8 (72.7)		5 (41.7)	7 (58.3)	
Bilharzial status						
Negative	18 (41.9)	25 (58.1)	0.344	15 (34.9)	28 (65.1)	0.365
Positive	19 (48.7)	20 (51.3)		16 (41)	23 (59)	
Grade						
Low	28 (57.1)	21 (42.9)	0.007	23 (46.9)	26 (53.1)	0.031
High	9 (27.3)	24 (72.7)		8 (24.2)	25 (75.8)	
Pathological stage						
Ta	9 (81.8)	2 (18.2)	0.037	9 (81.8)	2 (18.2)	0.133
T1	13 (68.4)	6 (31.6)		11 (57.9)	8 (42.1)	
T2	14 (48.3)	15 (51.7)		11 (37.9)	18 (62.1)	
T3	8 (34.8)	15 (65.2)		7 (30.4)	16 (69.6)	

Test of significance: Chi-square test. P-value < 0.05 is considered significant. TCC: transitional cell carcinoma.

SOX2 high expression was statistically significant with tumor grade as well as tumor pathological stage (P = 0.007 and 0.037, respectively). Low grade tumors were associated with negative/low SOX2 expression, whereas high grade tumors showed high SOX2 expression. Similarly, SOX2 expression became higher with increasing invasiveness of the tumor. Non-muscle invasive TCC (pTa-T1) showed negative/low SOX2 expression compared to muscle invasive TCC (pT2-T3) (P = 0.037). No significant associations were seen in relation to patients’ age, gender and bilharzial status.

Livin expression showed significant association with tumor grade (P = 0.031). The more increase of the tumor grade, the higher livin expression was. No significant associations were detected with livin expression and other clinicopathological factors including age, gender, bilharzial status and pathological stage.

### Expression of SOX2 and livin in SCC

Regarding SCC, the correlation between SOX2 and livin expressions and the clinicopathological data were summarized in Table 3.

**Table 3:** Relation of SOX2 and Livin Expressions With Clinicopathological Features of Bladder SCC (35 Cases)

Clinicopathological features	SOX2			Livin		
	Low (%)	High (%)	P-value	Low (%)	High (%)	P-value
Age						
≤ 50 years	5 (35.7)	9 (64.3)	0.583	6 (42.9)	8 (57.1)	0.206
> 50 years	7 (33.3)	14 (66.7)		5 (23.8)	16 (76.2)	
Gender						
Male	11 (36.7)	19 (63.7)	0.431	8 (26.7)	22 (73.3)	0.166
Female	1 (20)	4 (80)		3 (60)	2 (20)	
Bilharzial status						
Negative	3 (27.3)	8 (72.7)	0.424	1 (9.1)	10 (90.9)	0.058
Positive	9 (37.5)	15 (62.5)		10 (41.7)	14 (58.3)	
Grade						
GI	8 (66.7)	4 (33.3)	0.014	8 (66.7)	4 (33.3)	0.005
GII	3 (18.8)	13 (81.2)		2 (12.5)	14 (87.5)	
GIII	1 (14.3)	6 (85.7)		1 (14.3)	6 (85.7)	
Pathological stage						
Ta	0 (0)	0 (0)	0.555	0 (0)	0 (0)	0.144
T1	3 (42.9)	4 (57.1)		4 (47.1)	3 (42.9)	
T2	5 (26.3)	14 (73.7)		6 (31.6)	13 (68.4)	
T3	4 (44.4)	5 (55.6)		1 (11.1)	8 (88.9)	

Test of significance: Chi-square test. P-value < 0.05 is considered significant. SCC: squamous cell carcinoma.

SOX2 and livin expressions were significantly associated with tumor grade (P = 0.014 and P = 0.005, respectively). No correlation was found between SOX2 or livin expressions and other clinicopathological factors including age, gender, bilharzial status and pathological stage.

### Correlation between SOX2 and livin in primary urinary bladder carcinomas

Overall, the correlation observed between SOX2 and livin expression in primary urinary bladder carcinomas was significantly positive (P < 0.001). This was also maintained in TCC and SCC cases (P < 0.001 and P = 0.002, respectively).

### Prognostic value and survival analysis

As regards TCC, we found that the time of OS ranged from 6 to 60 months with a mean ± standard deviation (SD) of 26.27 ± 15.77 months and a median survival time of 20 months. The OS was 54.9%.

**Figure 3:**
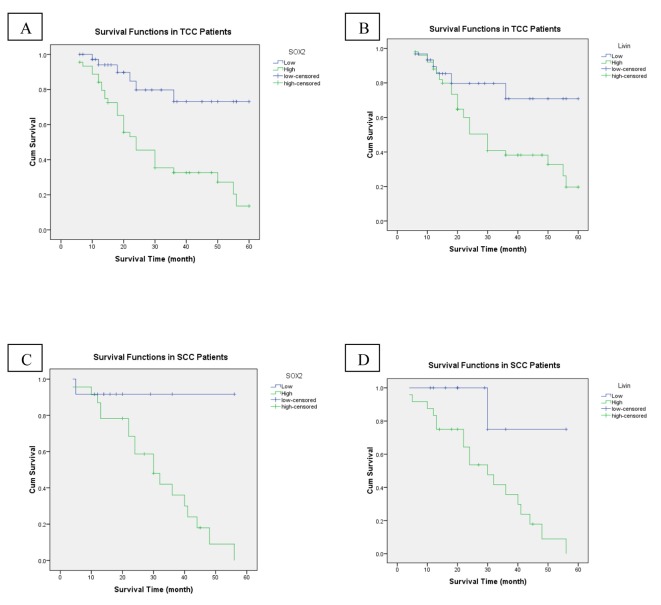
An analysis of overall-free survival in patients with bladder cancer by the Kaplan-Meier method. (A) According to SOX2 low/high expression in TCC patients (P < 0.001). (B) Low/high livin expression in TCC patients (P = 0.025). (C) SOX2 low/high expression in SCC patients (P = 0.041). (D) Low/high livin expression in SCC patients (P = 0.021).

High stage was the only adverse prognostic clinicopathological factor (P = 0.009). While OS rate was not significantly influenced by patients’ age (P = 0.512), sex (P = 0.432), grade (P = 0.126) and bilharzial status (P = 0.081). Compared to patients with high SOX2 expression, the patients whose tumor cells showed low expression of SOX2 had significantly better outcomes in OS (P < 0.001) (Fig. 3A). As regards livin expression, we found that high livin expression was significantly associated with shorter OS and low expression associated with better OS (P = 0.025) (Fig. 3B). In the multivariate analysis, high tumor stage and high SOX2 expression continued to be significant predictors of OS (P = 0.017, P = 0.015 respectively).

Regarding SCC, we found that the time of OS ranged from 4 to 56 months with a mean ± SD of 25.54 ± 13.77 months and a median survival time of 22 months. The OS was 45.7%.

High tumor grade and high stage were the adverse prognostic clinicopathological factors (P = 0.005, P = 0.048, respectively). While OS rate was not significantly influenced by patients’ age (P = 0.378), sex (P = 0.692), and bilharzial status (P = 0.780). Compared to patients with high SOX2 expression, the patients whose tumor cells showed low expression of SOX2 had significantly better outcomes in OS (P = 0.041) (Fig. 3C). As regards livin expression, we found that high livin expression was significantly associated with shorter OS and low expression associated with better OS (P = 0.021) (Fig. 3D). In the multivariate analysis, high tumor grade continued to be a significant predictor of OS (P = 0.033).

## Discussion

CSCs, a small subpopulation of cancer cells within tumors, have stem cell-like properties. CSCs have been identified in different cancer types [10-12, 24]. Although the CSC in bladder carcinoma has been frequently studied, the specific roles of bladder CSC are yet to be clarified [13]. These CSCs have been supposed to initiate carcinogenesis and have higher apoptosis resistance properties than those of differentiated cancer cells. These anti-apoptotic properties protect the CSCs from apoptosis stimuli and could lead to cancer resistance and recurrence. CSCs may be a promising strategy for cancer gene therapy [36].

SOX2 is over-expressed in CSCs and plays an important role in tumorigenesis and recurrence of multiple human cancers [22]. The function of SOX2 in apoptosis-resistant nature of cancer cells and its underlying mechanism still need to be explained.

Deficiency of apoptosis is related to tumorigenesis and cancer cell proliferation. The IAPs are antiapoptotic proteins which cause cancer cells resistant to apoptotic stimulation. Livin, a novel IAP family member, acts as an inhibitor of downstream caspases 3, 7 and 9 to cause apoptotic insensitivity [29].

Bladder cancer is the most common malignancy of the urinary tract, which has an increased morbidity and mortality all over the world. Tumor grade and stage have been identified as the most powerful prognostic factors for bladder cancer. Other clinicopathologic factors cannot fail to predict outcome accurately.

During the past decade, certain changes have been observed in the features of bladder cancer in Egypt, with significant increase in the incidence of TCC and decrease in SCC cases. Noticeable increases in the patients’ mean age with a dramatic decline in the incidence of associated schistosomiasis were reported [4, 37].

The current study showed a much higher frequency rate of TCC (70.1%) than SCC (29.9%) cases. Evidence of schistosomiasis was found in 53.8% of patients. A significantly lower incidence of associated schistosomiasis was noticed in TCC than in SCC cases. The mean age of SCC patients was significantly lower than those with TCC, with a male to female predominance in both types. The relatively younger patients’ age incidence in SCC than in TCC and the male predominance in both types were consistent with those mentioned in a previous report [37]. Notably, all SCC specimens included in this study were classified as muscle-invasive tumors, which point to the more aggressive nature of SCC. Regarding the survival analysis, patients with TCC had a better OS than those with SCC. The OS was significantly associated with tumor stage in TCC cases and with both tumor grade and stage in SCC cases. In multivariate analysis, high tumor stage was the predictor of OS in TCC patients (P = 0.017), while high tumor grade is the predictor of OS in SCC patients (P = 0.033). OS was not significantly affected by other clinicopathological factors in both TCC and SCC patients.

We observed that SOX2 and livin expressions were significantly higher in tumor tissues compared with their adjacent non-neoplastic tissues. SOX2 high expression was more in SCC cases (65.7%) than in TCC cases (54.9%). Furthermore, SCC cases showed higher positivity for livin than that detected in TCC cases (68.6% and 62.2%, respectively). However, such differences were not statistically significant (P = 0.189 and P = 0.330, respectively).

These results were consistent with previous studies in which SOX2 was over-expressed in esophageal, colorectal, breast and lung carcinomas [16, 17, 21, 22]. However, in gastric cancer, SOX2 was downregulated [38]. Also reports issued by Dai et al [30] and Gazzaniga et al [31] stated that livin was over-expressed in lung and bladder cancers, respectively.

In our study no correlation was found between SOX2 or livin expression and age and sex of both TCC and SCC patients. These findings coincided with previous results [39].

To the best of our knowledge, this study is the first to evaluate the expression of both SOX2 and livin in primary bladder carcinomas including TCC and SCC regarding bilharziasis status. We found no significant line of demarcation between bilharziasis associated and non-bilharziasis associated bladder carcinomas regarding their SOX2 and livin expressions suggesting that bilharziasis may have no significant role in up-regulation of either SOX2 or livin in TCC and SCC.

Also we are the first to rule out the expression of SOX2 in bladder SCC. We found that SOX2 expression was significantly correlated with tumor grade of both TCC and SCC (P = 0.007 and 0.014, respectively). In previous reports, SOX2 high expression was detected in high-grade more than in low-grade TCC [39]. It has been accepted that SOX2 expression was significantly associated with higher histological grade of esophageal carcinoma [16]. Accordingly, the results on the role of SOX2 in bladder cancer were coincided with that in other cancers.

We also noticed that SOX2 expression was significantly associated with pathological tumor stage of TCC, which indicated that SOX2 expression may be associated with progression in TCC. No positive association was found between SOX2 and pathological stage of bladder SCC. A role for SOX2 in cancer progression has been reported by several studies. SOX2 was found to be involved in invasion and metastasis of pancreatic intraepithelial neoplasia [15]. SOX2 expression was significantly correlated with lymph node metastasis and the stage of tumor invasion in gastric cancer [23]. Further analysis of the oncogenic function of SOX2 and underlying molecular mechanisms accounting for SOX2 being a marker for prognosis still remains to be highlighted.

Regarding the survival analysis, our investigation revealed that the expression of SOX2 in both TCC and SCC was correlated with poor clinical outcome (P < 0.001 and P = 0.41, respectively). Multivariate survival analysis demonstrated that SOX2 was an independent prognostic factor of outcomes in patients with TCC (P = 0.015). Consistently, most findings suggested that SOX2 expression could be a marker of poor prognosis in esophageal SCC, lung adenocarcinoma and oral tongue SCC [16, 40, 41].

To the best of our knowledge, this study is the first to analyze the expression of livin in bladder SCC. Our findings showed significant correlation between livin expression and tumor grade of both TCC and SCC (P = 0.031 and P = 0.005, respectively). This is in accordance with the results of Wang et al [27] who found a direct correlation between livin expression and higher-grade tumors. However, Gazzaniga et al [31] and Zhu et al [42] revealed a differential expression pattern of livin in bladder tissues.

In our study, livin expression was proved to be higher in MIBC (pT2-pT3) than that in non-MIBC (pTa-pT1) but results did not reach a significant level. Similarly, Zhu et al [42] found no significant difference in livin expression between non-muscle infiltrating TCC and muscle infiltrating TCC.

Concerning the survival analysis, we found that high expression of livin in both TCC and SCC was significantly correlated with poor OS (P = 0.025 and P = 0.21, respectively). According to these results, we emphasize that high livin expression acts as a powerful prognostic factor in bladder cancer progression.

In conclusion, we found a significant association between SOX2 and livin expressions in primary bladder cancers. SOX2 up-regulates the expression of livin which inhibits apoptosis. Nevertheless, the overexpression of SOX2 elevates livin expression. Knockdown of SOX2 may be used as a tool to decrease livin expression, thereby initiation of apoptosis related caspases activation. SOX2/livin pathway regulates CSC survival, so it could be targeting as an effective therapeutic strategy for cancer treatment.
